# Genome-Wide Footprints of Pig Domestication and Selection Revealed through Massive Parallel Sequencing of Pooled DNA

**DOI:** 10.1371/journal.pone.0014782

**Published:** 2011-04-04

**Authors:** Andreia J. Amaral, Luca Ferretti, Hendrik-Jan Megens, Richard P. M. A. Crooijmans, Haisheng Nie, Sebastian E. Ramos-Onsins, Miguel Perez-Enciso, Lawrence B. Schook, Martien A. M. Groenen

**Affiliations:** 1 Animal Breeding and Genomics Centre, Wageningen University, Wageningen, The Netherlands; 2 Department of Animal Science and Food Technology, Universitat Autonoma de Barcelona, Bellaterra, Spain; 3 Animal Science Department, Centre for Research in Agricultural Genomics, Bellaterra, Spain; 4 Life and Medical Sciences, Institució Catalana de Recerca i Estudis Avançats, Barcelona, Spain; 5 Institute for Genomic Biology, University of Illinois, Urbana, Illinois, United States of America; University of Uppsala, Sweden

## Abstract

**Background:**

Artificial selection has caused rapid evolution in domesticated species. The identification of selection footprints across domesticated genomes can contribute to uncover the genetic basis of phenotypic diversity.

**Methodology/Main Findings:**

Genome wide footprints of pig domestication and selection were identified using massive parallel sequencing of pooled reduced representation libraries (RRL) representing ∼2% of the genome from wild boar and four domestic pig breeds (Large White, Landrace, Duroc and Pietrain) which have been under strong selection for muscle development, growth, behavior and coat color. Using specifically developed statistical methods that account for DNA pooling, low mean sequencing depth, and sequencing errors, we provide genome-wide estimates of nucleotide diversity and genetic differentiation in pig. Widespread signals suggestive of positive and balancing selection were found and the strongest signals were observed in Pietrain, one of the breeds most intensively selected for muscle development. Most signals were population-specific but affected genomic regions which harbored genes for common biological categories including coat color, brain development, muscle development, growth, metabolism, olfaction and immunity. Genetic differentiation in regions harboring genes related to muscle development and growth was higher between breeds than between a given breed and the wild boar.

**Conclusions/Significance:**

These results, suggest that although domesticated breeds have experienced similar selective pressures, selection has acted upon different genes. This might reflect the multiple domestication events of European breeds or could be the result of subsequent introgression of Asian alleles. Overall, it was estimated that approximately 7% of the porcine genome has been affected by selection events. This study illustrates that the massive parallel sequencing of genomic pools is a cost-effective approach to identify footprints of selection.

## Introduction

Animal domestication is the process whereby animals adapt to humanized environments through a process of selection resulting in a different phenotype of the domestic animal compared to its wild counterpart [1].

Artificial selection is perhaps the best understood aspect of the domestication process [2]. Understanding how domestication has shaped the patterns of genetic variation is important, since domestication can reflect rapid evolution triggered by human-generated pressures. Previous studies have shown that domestication was associated with selective pressures on specific genes related to eg. growth [3] and coat color [4], [5] and that artificial selection might have contributed to reduce polymorphism levels and increase linkage disequilibrium in some domesticated species [6]–[9]. However, the degree to which adaptive evolution has affected DNA polymorphism genome wide has not been extensively studied and few studies have determined which types of selection are most prevalent in domesticated animals.

The domestication of the European wild boar started around 13,000 B.P. and most likely was triggered by the introduction of domestic pigs by Middle East Neolithic farmers [10]. The process of selection for different environments within the European continent might have resulted in the generation of a wide variety of domestic pig breeds with divergent phenotypes [11]. Behavioral phenotypes would also have been modified due to changes in social structure, reproduction and adaptation towards humans [1]. Today most breeds are created in captivity but, during the beginning of domestication, selected animals would freely breed among human settlements and would have had a flexible diet [12], [13] resembling extensive pig production systems which still exist today (eg. Iberian pig). Thus, pig domestication can be defined as a mixture of weak selection (applied postzygotically with no conscious wish to alter a breed) and strong selection (applied prezygotically guided by a predetermined goal) for favorable traits [14], [15]. Nowadays, a small number of breeds dominate pig production worldwide, i.e. Large White, Landrace, Duroc and Pietrain which have distinct phenotypes generated by selection applied with different intensities for desired production attributes.

Genomic regions under selection are expected to display extreme levels of DNA polymorphisms in comparison with unselected regions. Such regions displaying footprints of selection can be studied by determining the allelic variation of SNPs (single nucleotide polymorphisms). In human populations, previous studies have revealed footprints of recent positive selection involving genes linked to resistance to malaria [16], dairy farming [17], and brain development [18]. Likewise, studies have detected signatures of recent balancing selection in humans in response to disease [19] and to the need for kin recognition and survival [20]. However, these studies were based on SNP genotypes that had initially been identified by an ascertainment (or SNP discovery) process. This ascertainment creates a bias due to the fact that the sample size used for the SNP discovery panel is often small, causing a frequency-specific distortion in SNP discovery. Consequently, the frequency spectrum obtained from the two-tier sampling differs from the frequency spectrum obtained by sequencing the entire study sample [21]. This will affect any statistical measures that rely on the site frequency spectrum, including nucleotide diversity, Tajima's D and *F_st_*. Alternatively, massive parallel sequencing (MPS), also called next generation sequencing, is considerably less expensive and is able to produce sequence data orders of magnitude higher than can be obtained by traditional Sanger capillary sequencing [22], [23]. Consequently, MPS provides the opportunity to obtain genome-wide estimates of the genetic diversity of a species without the effect of ascertainment bias.

Previously, within a large scale SNP discovery project [24], a large data set of ∼380 million sequences were generated from pooled reduced representation libraries (RRLs) of Large White, Landrace, Duroc and Pietrain and the wild boar using the Genome Analyzer (GA, Illumina). Here we investigated whether signatures of selection due to domestication or recent breeding practices can be detected using this data set. We identified genomic regions with extreme values of nucleotide diversity and with extreme values of genetic differentiation among pig populations. Statistics were developed to characterize nucleotide diversity and genetic differentiation in pooled GA sequence data generated using MPS.

Our results suggest a prevalence of positive selection in genomic regions known to harbor genes affecting coat color, behavior, muscle development, and metabolism following domestication. Furthermore, these results indicate that the olfactory receptors and the immune system (MHC genes) have most likely undergone a process of balancing selection both in the domestic European pig breeds and in the wild boar.

## Results

### Sequence analysis

**Figure 1:**
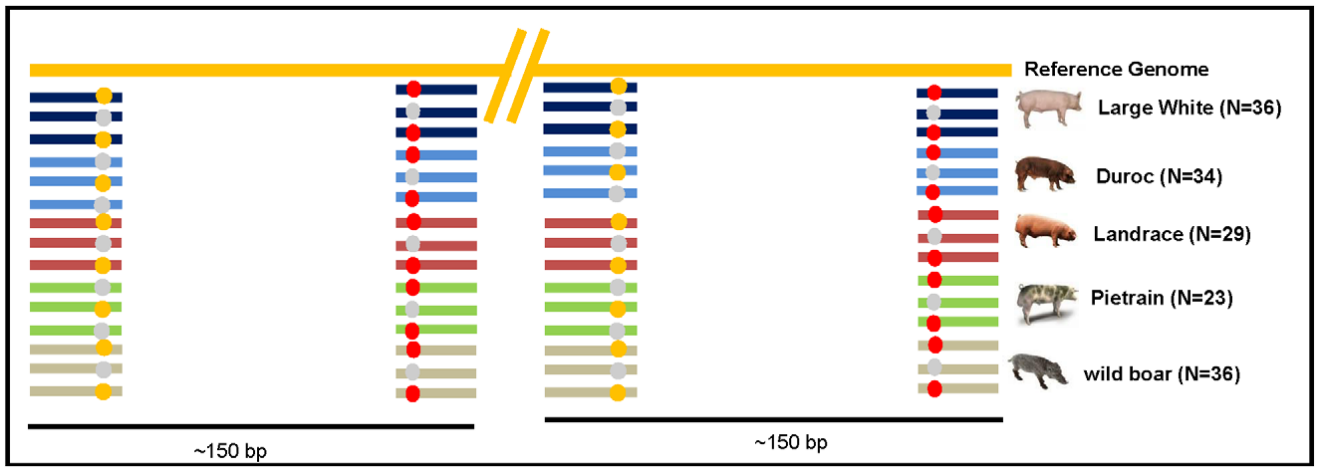
Schematic drawing showing the expected alignment between reads and the reference genome. The colored bars represent the reads; each color corresponds to a different pig population. The reads originated from ∼150 bp fragments of RRL libraries of pooled DNA for each pig population. Therefore, for each read the identification is only available at the population level. Colored dots on the reads represent SNP positions.

Approximately 380 million GA sequences (hereafter referred to as ‘reads’) were generated from RRLs obtained from pooled DNA of five pig populations (Duroc, Landrace, Large White, Pietrain and the wild boar; hereafter referred to as ‘sampled populations’) with divergent phenotypes (Figure 1; Table S1). The sampled animals were non-related (no shared grandparents) and represent current global breeds. Raw reads were preprocessed to remove reads with errors (see Methods) and the remaining 200 million reads were aligned with the pig genome reference assembly (Build 8). Since reads were obtained from RRLs of pooled DNAs, reads were aligned to the reference genome, forming clusters, for which only the information of the population of origin was available (Figure 1). A total of approximately 2% of the porcine genome met alignment quality parameters (see Methods), and the average sequencing depth ranged from 7.5X for wild boar to 10X for Duroc (Table 1). SNPs identified included 70% transitions and 30% transversions (Figure S1) and rare variants (SNPs observed in only one read) were nearly absent (Figure S1). The correlation between the GC content and the total number of aligned bases per cluster ranged between 0.67 and 0.72 (*p*-value <0.0001) for all sampled populations.

**Table 1:** Summary statistics of sequence filtering and alignment of overall chromosomes in pig breeds and in wild boar.

Population	Total raw sequences	Total filtered sequences	Total aligned length	Average Sequence depth
**Large White**	81,501,174	45,233,951	40,580,400	7.8
**Landrace**	88,002,147	43,341,083	42,832,864	8.3
**Pietrain**	71,561,104	42,242,606	51,566,246	9.7
**Duroc**	75,925,390	36,424,674	37,176,400	10.0
**wild boar**	65,053,290	28,723,892	29,229,683	7.5

### Genome-wide estimates of nucleotide diversity in pig

**Figure 2:**
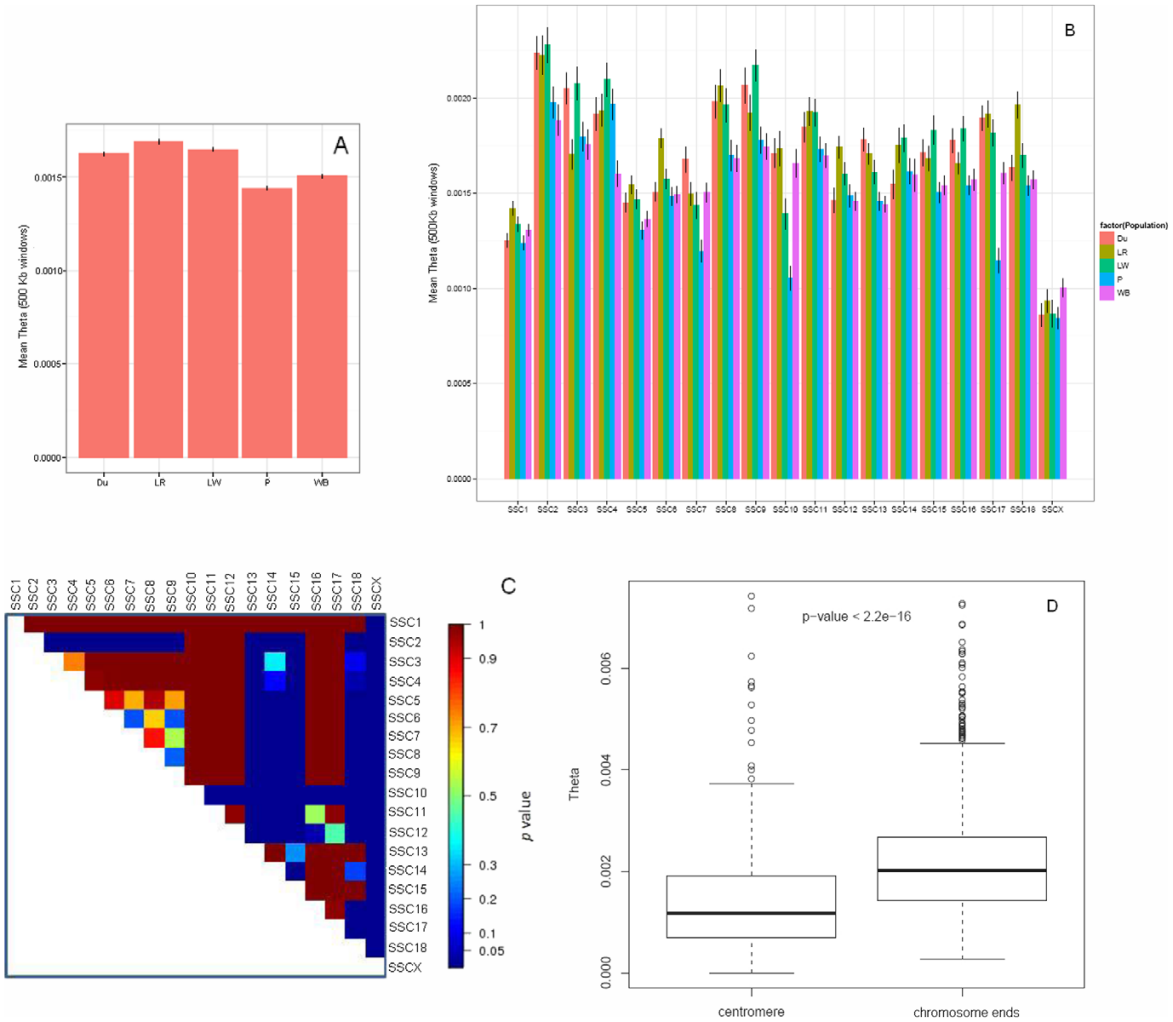
Average nucleotide diversity per chromosome and sampled population. Nucleotide diversity was estimated in non-overlapping 500 Kb windows (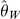). A- Average 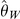 overall chromosomes per population. B- Average 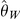 per chromosome for each population. Vertical lines represent standard errors of the mean. C- Heatmap representing the *p*-values obtained from comparing the average 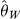 per chromosome. D- Boxplot showing that centromeres and chromosome ends behave different in terms of nucleotide diversity.

**Figure 3:**
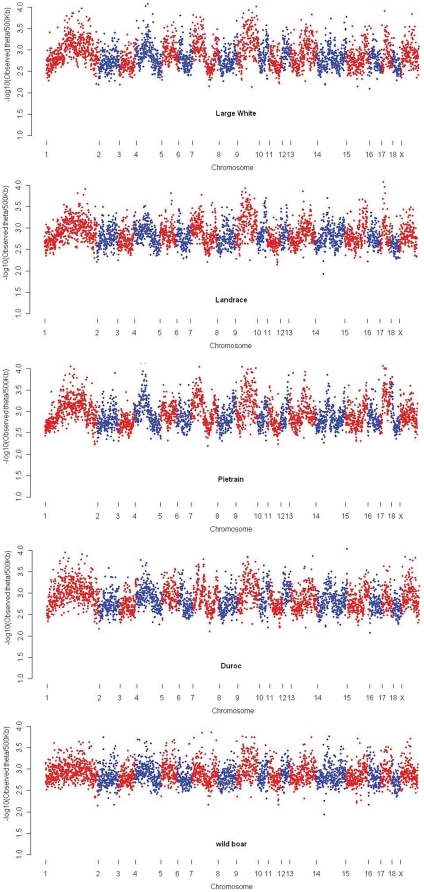
Genome-wide variation of nucleotide diversity in the studied populations. Each dot represents the observed nucleotide variation over a window of 500 Kb.

The Watterson's estimator of nucleotide diversity (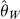) was modified in order to take into account important characteristics of the data (pooled DNA, sequencing errors and the lack of rare variants). Nucleotide diversity was estimated in non-overlapping windows of 500 Kb for each chromosome and for each population. The Landrace breed displayed the highest average of 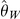 over all the chromosomes, whereas the Pietrain breed displayed the lowest average of 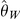 (Figure 2A). The average of 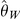 per chromosome is shown for each population in Figure 2B. In particular, the average of 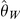 for the Pietrain breed on SSC8, SSC15, and SSC18 was significantly lower (*p*-value <0.0001) than the average of 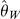 observed in the Landrace and Large White breeds (Figure 2B). Considering the comparison of the average of 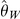 between chromosomes, significant differences (*p*-value<0.05) were also observed between autosomes (Figure 2C). A significant decrease of 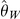 (*p*-value<0.0001) was observed on SSC X (mean 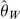 = 0.0008) compared to the autosomes (0.0010<mean 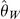 <0.022) (Figure 2C). For metacentric and submetacentric chromosomes the values of 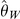 were significantly higher at the chromosome ends than at the centromeres (Figure 2D). The variation of 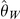 along the chromosomes was similar across all populations (Figure 3).

### Identification of candidate regions of recent selection

In order to identify outlier estimates of 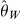 potentially representing candidate regions that might have been under selection, 95% confidence intervals (CI) of 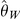 were obtained for each window by performing neutral coalescent simulations with recombination (see Methods). Genomic regions with 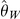 values outside the boundaries established by the CI were classified as candidate regions for recent selection. The number of candidate regions putatively under selection identified in the different populations is summarized in Table 2. The Pietrain breed showed the highest number of regions with a significant low value of 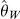 whereas the wild boar had the lowest. Approximately 70% of putatively selected regions were observed in only one of the sampled populations (Table 2).

**Table 2:** Number of regions per breed with significant values of nucleotide diversity (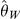).

Breed	TWHT	TWLT	LW	LR	P	Du	WB
**Large White (LW)**	391	317		124 (39)	119 (38)	88 (28)	32 (10)
**Landrace (LR)**	446	354	155 (35)		122 (34)	87 (25)	34 (10)
**Pietrain (P)**	478	408	164 (34)	200 (42)		86 (21)	32 (8)
**Duroc (Du)**	421	331	137 (33)	150 (36)	147 (35)		27 (8)
**wild boar (WB)**	226	111	91 (40)	100 (44)	107 (47)	90 (40)	

TWHT- total number of windows with a significant high value of 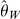. TWLT- total number of windows with a significant low value of 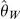. Values shown above the diagonal are the number of regions with low 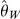 values that were shared between sampled populations (parentheses enclose the percentage, calculated over the TWLT between the pairs compared); values shown below the diagonal are the number of regions with high 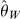 values that were shared between sampled populations (the percentage in parentheses is calculated over the TWHT between the pairs compared).

**Figure 4:**
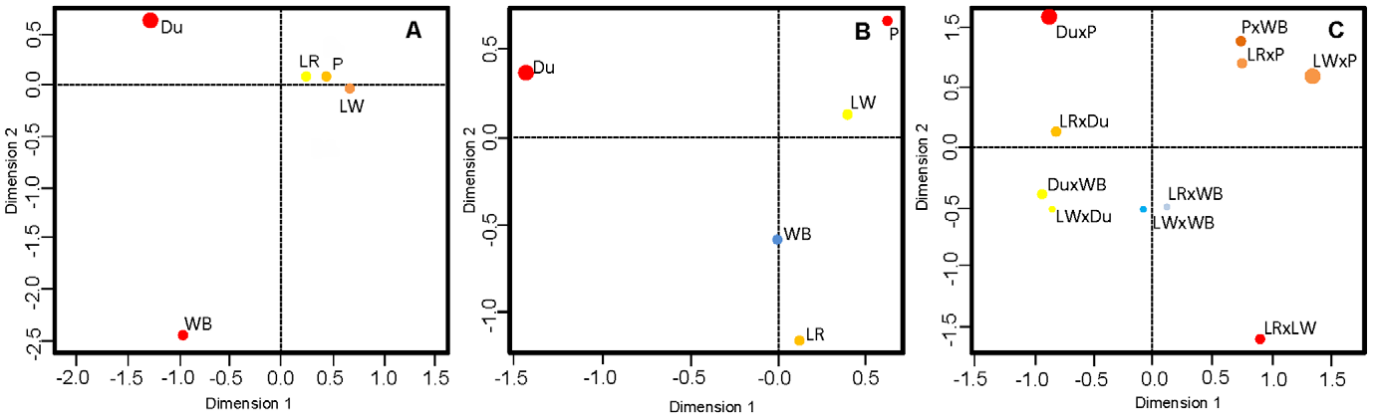
Correspondence analysis of population *vs.* genomic regions under selection and observed 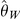. A- LT regions (regions with 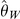 smaller than the lowest 95% C.I. boundary); B- HT regions (regions with 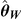 larger than the highest 95% C.I. boundary); C- Genomic regions with significant high *F_st_* values. Different color and point size indicate the relative contribution of each population to the space arrangement in the plot. Brown color and larger size indicates highest contribution, light blue and smaller size indicates smallest contribution.

Approximately 36% of the candidate regions with a value of 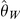 below the lowest C.I. boundary (LT) were shared among the white coated breeds, Pietrain, Large White, and Landrace. The Duroc (red coated) breed shared fewest regions with the other breeds (Table 2). Only 10% were shared between any of the domesticated breeds and the wild boar population (black coated) (Table 2). The candidate regions under selection that were shared between populations were also investigated through correspondence analysis. The white coat color breeds Landrace, Large White and Pietrain share more candidate regions under selection and therefore cluster together (Figure 4A). The Duroc breed apparently has undergone a divergent effect of positive selection when compared with the other breeds since this breed contributed most to the space arrangement of the plot shown in Figure 4A.

Approximately 40% of the candidate regions with values of 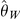
above the highest C.I. boundary (HT) were shared among the domestic breeds but only 20% were shared with the wild boar population. The correspondence analysis of the HT regions in all the breeds and the wild boar showed that the wild boar shared many regions with the white breeds (Figure 4B). The Duroc, Landrace, and Pietrain breeds represented the domestic breeds that contributed most to the spatial arrangement of the plot (Figure 4B) and the ones that shared the lowest number of HT regions.

### Genes overlapping candidate regions of selection

After comparing the genomic locations of the identified candidate regions of recent selection with the available annotation of the pig genome, genes were identified which putatively have been under selection due to pig domestication (Table S2).

**Figure 5:**
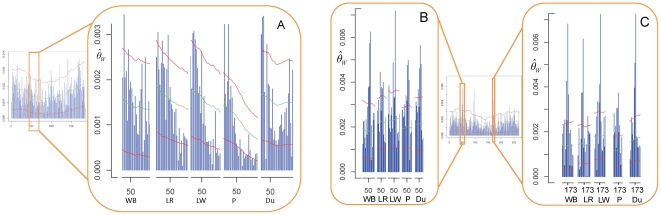
Variation of 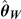 along the wild boar SSC8 and SSC7. Blue bars represent point estimates for each 500 Kb window. Red lines represent confidence interval limits with a significance level of 95%. Green lines represent the average 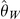 per window. The insets are enlargements of the orange boxes that show details of the variation of 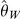 in genomic regions that deviate from the standard neutral model in all sampled populations. A- Detail of genomic regions with a significantly low 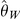 and that potentially contains the *KIT* gene. B- Detail of genomic regions with a significantly high 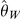 and that potentially contains the *TRIM26* gene member of the *SLA* locus. C- Detail of genomic regions with a significantly low 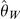 and that potentially contains the *OR4K13* gene.

We have identified LT regions overlapping with the *KIT* gene, responsible for the white color, on SSC8 in the Large White, Landrace, and Pietrain breeds, but not in the red coated Duroc breed or the black coated wild boar (Table 3; Figure 5A). A systematic overlap of LT regions with genes related to growth and muscle development (eg. *MAPK1* gene - SSC 14:51.5–52 Mb*)* was observed in domestic pig breeds (Table 3). Moreover, a consistent pattern was observed in which genes related with brain development and neuronal functions (eg. *PPP1R1B* gene - SSC12:15–15.5 Mb) overlapped LT regions in the domestic pig breeds but not in the wild boar population (Table 3; Table S2). In HT regions, we have detected several genes from the olfactory receptor complex and from the *SLA* locus located in SSC7, where the strongest signals correspond to the *TRIM26* gene (zinc finger protein 173) (Table 3; Figure 5A) and to the *OR4K13* gene (olfactory receptor 4K13) (Figure 5B). The *TRIM26* gene from the *SLA* locus is located between positions 24–24.5Mb on SSC7 and significantly high values of 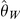 were observed in the Duroc, Pietrain, Landrace and Large White breeds. The *OR4K13* gene, from the olfactory receptor gene family overlaps with the genomic region between 86–86.5 Mb and displayed significant high values of 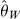 in the studied populations.

**Table 3:** Examples of genes putatively under selection.

	Population	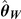	95% C.I.
*KIT*	Large White	0.00034	0.0005–0.002
	Landrace	0.00021	0.0006–0.002
	Pietrain	0.00025	0.0004–0-001
	Duroc	0.00067	0.0005–0.002
	wild boar	0.00078	0.0004–0.002
*TRIM26*	Large White	0.00487	0.001–0.004
	Landrace	0.00446	0.001–0.003
	Pietrain	0.00341	0.001–0.003
	Duroc	0.00370	0.001–0.003
	wild boar	0.00289	0.0006–0.003
*OR4K13*	Large White	0.00813	0.0008–0.003
	Landrace	0.00615	0.0006–0.002
	Pietrain	0.00340	0.0005–0.002
	Duroc	0.00863	0.0007–0.003
	wild boar	0.00681	0.0003–0.002
*MAPK1*	Large White	0.00029	0.0005–0.002
	Landrace	0.00043	0.0008–0.002
	Pietrain	0.00063	0.0006–0.002
	Duroc	0.00055	0.0004–0.002
	wild boar	0.00644	0.0003–0.002
*PPP1R1B*	Large White	0.00080	0.0011–0.003
	Landrace	0.00065	0.0011–0.0027
	Pietrain	0.00119	0.0012–0.0026
	Duroc	0.00057	0.0010–0.0027
	wild boar	0.00073	0.0007–0.0026
*LRRTM2*	Large White	0.00137	0.0014–0.0040
	Landrace	0.00098	0.0014–0.0038
	Pietrain	0.00105	0.0015–0.0035
	Duroc	0.00167	0.0012–0.0037
	wild boar	0.00153	0.0009–0.0036

Values represent the 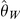 on the window overlapping the gene and the respective 95% confidence interval.

### Widespread signals of breed differentiation

Genomic regions affected by selection can also potentially be identified as outliers in the extreme tails of the empirical distribution of a measure of genetic differentiation, e.g., *F_ST_*. Such an approach has the advantage of being free of theoretical predictions regarding population structures or demography [25]. To this end, the measure *F_ST_* as defined by Nei [26] was adapted to accommodate the characteristics of the data, namely reads of pools, ascertainment bias against rare variants and sequencing errors. The estimates of *F_ST_* varied along chromosomes (Figure S2). Most *F_ST_* values were significantly different from 0 (*p*-value<0.05) (Figure S2) displaying an overall mean of 0.122 with a standard deviation of 0.187. The *F_ST_* values for all pair-wise population comparisons are shown in Table 4. The Duroc breed displayed the highest *F_ST_* value compared to the other breeds, having the largest divergence with the Pietrain. The Landrace and Large White breeds were the populations most similar to the wild boar. Genomic regions that displayed high genetic differentiation between populations - *F_ST_* values in the 95% quartile with significant *p*-values (<0.05) – represented candidate regions of positive selection and were selected for further analysis. A correspondence analysis was performed on these identified genomic regions in order to investigate whether the studied pairs of populations shared these regions (Figure 4C). The Landrace *vs.* wild boar and Large White *vs.* wild boar clustered together. In contrast, the Pietrain *vs.* Duroc clustered far apart from the other populations *vs.* Pietrain.

**Table 4:** Genetic differentiation between sampled populations.

	LW	LR	P	Du
**Large White (LW)**				
**Landrace (LR)**	0.10 (0.003)			
**Pietrain (P)**	0.10 (0.003)	0.12 (0.003		
**Duroc (Du)**	0.14 (0.003	0.14 (0.003)	0.16 (0.003)	
**wild boar (WB)**	0.10 (0.003	0.11 (0.003)	0.13 (0.003)	0.13 (0.003)

Values represent the average genetic differentiation (F_st_) between population pairs (parentheses enclose standard errors of the mean).

### Types of biological processes under selection

To investigate whether certain GO categories or KEGG pathways were enriched in the identified candidate regions of recent selection, a gene set enrichment analysis was performed. Genes overlapping HT and LT regions were classified using gene ontology (GO) categories and functional pathway (KEGG) categories. If a genomic region contained multiple genes for the same GO/KEGG category, this category was only counted once thus avoiding GO/KEGG categories being significant due to a cluster of genes from the same gene family.

The results suggest an overrepresentation of genes related to muscle development, growth and melanogenesis in LT regions of domestic pig breeds and overrepresentation of genes related to immune defense in the wild boar (raw *p*-value<0.05) (Table S3). Furthermore an overrepresentation of genes related to metabolism was observed in the LT regions of all the studied populations (raw *p*-value<0.5). These results however were no longer significant after correcting for multiple testing (0.3<adjusted *p*-value<0.9). Within the HT regions, there was a significant overrepresentation (adjusted *p*-value<0.0001) of genes related to olfaction and other sensorial capabilities (Table 5). Within genomic regions extremely differentiated between the Duroc and Pietrain breeds, a significant enrichment of genes related to olfaction (adjusted *p-*value = 2×10^−3^) was observed. In fact, from a total of 1,577 genes located within the HT regions for all the studied populations, 144 genes encode for olfactory receptors.

**Table 5:** Enrichment of Gene Ontology (GO) categories and Kyoto Encyclopedia of Genes and Genomes (KEGG) pathways among HT regions.

GO category	Landrace	Large White	Pietrain	Duroc	Domesticated breeds	Wild boar
Sensory perception of smell	<0.0001	<0.0001	<0.0001	<0.0001	<0.0001	<0.0001
G-protein, coupled receptor protein signaling pathway	0.03	0.04	<0.0001	0.0008	0.02	<0.0001
G-protein, coupled to cyclic nucleotide second messenger signaling	ns	0.05	<0.0001	ns	0.04	ns
neurological system process	0.009	0.003	0.01	0.0008	<0.0001	ns
sensory perception	0.004	0.001	0.02	0.002	<0.0001	<0.0001
response to stimulus	0.003	<0.0001	0.0009	0.002	<0.0001	0.0001
**KEGG Pathway**						
Olfactory transduction	<0.0001	<0.0001	<0.0001	<0.0001	<0.0001	<0.0001

The analysis was performed for all sampled populations individually and for the domestic breeds in compound.

Values represent *p*-values for significance of enrichment, after correction for multiple testing.

“ns” indicates that the *p*-value was >0.05 (not significant).

## Discussion

By performing MPS of RRLs obtained from DNA pools we identified candidate regions within the porcine genome that putatively have been under selection. MPS on RRLs was shown to be a simple, cost-effective approach applicable to any species, even in the absence of a complete reference genome. Using a coalescent-based estimation of the uncertainty of the observed nucleotide diversity we have identified regions with a value of 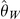 deviating significantly from the expectations and which are therefore candidate regions of selection. Genomic regions that had a 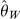 value significantly smaller than the expectations represented regions where the frequency of the favored allele in a haplotype with low diversity could have increased very rapidly and thus were considered as potential candidate regions of recent positive selection. In contrast, in genomic regions with a 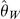 value significantly larger than the expectations an increase of variability was observed along with an increase of intermediate allele distributions and these regions were therefore considered as potential candidate regions for the occurrence of balancing selection [27]. Because demographic processes can mimic selection, the interpretation of outlier values of nucleotide diversity is not trivial. In fact, the occurrence of population growth can also result in a high occurrence of low-frequency alleles and population subdivision also results in the high occurrence of intermediate-frequency alleles. Furthermore, the occurrence of copy number polymorphisms can also result in the increase of nucleotide diversity [27], [28]. Therefore, throughout the discussion of our results the identified outlier regions will always be referred as candidate regions of selection. Nevertheless, this study provides the first genome-wide map of putative signatures of selection in the porcine genome. These results, together with knowledge regarding the functional aspects of the regions, provide new insights into the molecular nature of animal domestication and selection.

### Variation of nucleotide diversity between pig breeds and wild boar

Overall, and in agreement with previous studies [29], the Pietrain breed demonstrated the lowest 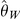 which is indicative of a small effective population size compared to other breeds and a low contribution to the genetic diversity of the *Sus scrofa* species [30]. It is worth noting that the wild boar population had low values of 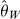 as compared to the Large White, Landrace, and Duroc breeds. This low level of 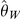 in the wild boar may be attributed to the severe decline of the European wild boar populations that over time might have lead to high levels of linkage disequilibrium and also to a smaller effective population size [6]. As reported previously for other species, we observed lower 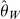 on SSCX compared to the autosomes, probably reflecting the smaller effective population size for this chromosome [31]. The observed significant increase of 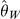 in the chromosome ends in comparison to the centromeric areas is consistent with previous studies in humans [32], [33] and other mammals [32], [34] and most likely is due to the higher level of recombination rate towards telomeres.

### Signatures of positive selection and genetic differentiation between breeds

Most of the putative signatures of positive selection were observed in a single population and, the number of candidate regions detected in domestic breeds was larger than in wild boar. Interestingly, domestic pig breeds shared a higher proportion of these regions between themselves than with the wild boar population. This suggests that domestication, or modern artificial selection, could have affected genes related to the same biological pathways in the different pig populations. The correspondence analysis of these genomic regions, suggests strong directional selection that is consistent with the history of pig breeds. Landrace and Large White clustered together, sharing many of the regions putatively under positive selection showing the lowest levels of genetic differentiation between breeds. In fact, the Landrace breed originated in Denmark from a cross between the wild boar and Large White [11]. In contrast, the Duroc shared less putative regions of positive selection with the other breeds and was the most genetically differentiated from the other breeds. This finding is consistent with previous studies [35], [36] adding support to the hypothesis that this breed has a distinct origin from the rest of European breeds. Historical records suggest that the Duroc breed originated from European red breeds or the Red Guinea Hog [35]. Our results revealed that genetic differentiation was higher between domestic breeds than between a given domesticated breed and the ancestor wild boar. Furthermore, the analysis of clustering of regions with high level of genetic differentiation revealed a pattern, which is in accordance with the history of breed domestication and selection. These findings are compatible with the occurrence of multiple domestication events in Europe, resulting in the generation of several domestic pig breeds that are more similar to its ancestor and yet are highly differentiated in terms of phenotypes and genotypes. An alternative explanation is the introgression of Asian germplasm in a post-domestication stage. In fact, the use of a pig from Canton province in China for the creation of the Large White pig has been documented [11], and the introgression of Asian alleles has been observed in previous genetic studies [6], [37]. This may explain the high levels of nucleotide diversity in the Large White and Landrace breeds.

### Footprints of selection reveal genetic differences in coat color, growth, and behavior due to domestication

Since the dataset comprised approximately 2% of the genome, windows of 500 Kb were used in order to have a reasonable number of SNPs per window hence limiting the resolution of the gene set enrichment analysis. In addition, the current annotation of the pig genome has limited availability of GO terms and KEGG pathways further reducing the sensitivity of the analysis. Because of these limitations we were only able to provide suggestive evidence for over representation of specific biological processes affected by positive selection. The biological functions of genes located within the genomic regions that were putatively under positive selection, suggests that recent positive selection occurred in the domestic pig breeds in genes associated with coat color, behavior, growth, and muscle development. Concerning coat color, the genomic region containing the *KIT* gene was shown to have unusually low diversity in the white breeds -Large White, Landrace, and Pietrain - but not in the Duroc and wild boar populations. This observation is in agreement with the identification of a mutation in the *KIT* gene present in all white breeds and absent in breeds with a colored coat [4] providing support for our method. These results also revealed that genes involved in growth and muscle development overlap with regions putatively under positive selection and with regions with significantly high levels of genetic differentiation in domestic pig breeds. This observation is consistent with the history of the domestic pig breeds studied, which have higher growth rates and proportion of muscle than the ancestral wild boar populations. A genome scan in chicken revealed a similar pattern – several genes related to growth, muscle development and skin color (yellow skin) were identified as having been under positive selection [38]. In the chicken it was shown that a mutation in the gene that encodes for the thyroid hormone might have arisen during domestication of chicken, with functional consequences in terms of metabolism and growth of the domestic chicken populations. Our results also provide suggestive evidence for the selection of genes linked to metabolism that likely reflects selection due to adaptation to human-altered environments and feed. Our results and those of Rubin and colleagues [38] are consistent with the evolution for size and coat color being a consequence of domestication and not a consequence of natural selection or genetic drift. Interestingly, the wild boar populations revealed evidence of positive selection in genes related to disease resistance. This observation may reflect natural selection for survival and fitness in the wild and suggests that the release of selective constrains in genes related to disease resistance in domestic breeds could have lead to a faster accumulation of genetic diversity among these genes.

The process of domestication can also result in related behavioral changes. Behaviors that are important for survival in nature, like finding food and predator avoidance, may not provide significant advantages in humanized environments associated with captive breeding [1]. Moreover, it is expected that farmers would have selected for more docile animals. In this study, we observed a systematic pattern in which genes related to brain and neuron functions overlapped regions putatively under positive selection in the domestic pig populations but not in the wild boar population. For example, one region harbored the *PPP1R1B* gene that codes for the dopaminergic neurotransmitter critical for motivated behavior, working memory, and reward-related learning [39]. Therefore we propose that similarly to dogs which have inferior observational learning skills compared to wolves [40], these signatures might have been a consequence of domestication.

### Are the MHC and the olfactory receptors under the influence of balancing selection?

Several genes identified in this study have been shown to be under the influence of balancing selection in other mammals. Our results indicate the maintenance of unusually high nucleotide diversity in the MHC genes of the porcine genome, suggesting an effect of balancing selection similar to observations in other mammals such as e.g., dogs [41], cattle [42], sheep [43], rat [44], rhesus macaque [45], and humans [19]. The overrepresentation of olfactory receptor genes and of genes related to other sensory traits of the pig were significant in our analysis (*p*<0.001). The maintenance of high variability in olfactory receptors has been observed in humans [20], other primates [46] and mouse [47]. In humans, a model of overdominance has been proposed for the evolution of olfactory receptors [20]. Individuals that are heterozygous for olfactory receptors can potentially double the number of different odorant-binding sites encoded in the genome, thus allowing the individual to discriminate among closely related structural odorants [48]. Our results suggest that individual recognition in pigs is crucial for survival not only for the wild boar reared in wild environments but also for domestic pigs in human-altered environments.

### Conclusions

Artificial selection has produced dramatic alterations in livestock environments, and undoubtedly has left important selective footprints throughout the genomes of domesticated species. To date, research of livestock has been centered on the identification of individual genes as candidates for selection. This study provides a genome-wide characterization of DNA polymorphism of the pig and yields important insights into the types of biological processes that have been targets of selection during pig domestication. Putative signals of selection were detected for coat color, growth and muscle development. Furthermore, our results suggest that selection might also have occurred at genes related to metabolism, behavior, olfaction and disease resistance. This investigation showed that the study of domestication can progress rapidly due to the use of massive parallel sequencing proposing a top-down approach, where candidate genes can be identified in a whole-genome approach by using a representative sample of the genome.

## Methods

### Material

We analyzed a total of 380 million 36 bp reads from four pig breeds, including Duroc (N = 34), Landrace (N = 29), Large White (N = 36), Pietrain (N = 23) and from the wild boar (N = 36) (Figure 1; Table S1). Reads were generated from RRL libraries produced from DNA pools of each of the breeds. Details concerning DNA extraction, preparation of DNA pools and RRL libraries have been described previously [24]. RRL libraries were sequenced using the 1G Genome Analyzer (Illumina, San Diego, California, USA).

### Sequence analysis and estimation of nucleotide diversity

For each breed, reads were trimmed at 33 bp and reads with homopolymers (>17nt), unknown bases and overrepresented sequences (more than two times the observed average frequency) were removed. We also applied filters for sequence quality based on the quality scores provided by the Illumina base calling software. Because base quality decreases towards the 3′end of the reads [34], reads with an average quality lower than 20 were also removed. The remaining reads were aligned to *Sus scrofa* assembly 8 (ftp://ftp.sanger.ac.uk/pub/S_scrofa/assemblies/PreEnsembl_Sscrofa8) allowing up to two mismatches using MAQ (49). For each aligned base of the consensus sequence, the alignment algorithm generates [49] a quality score (*pSNP*). This measure *pSNP* quantifies base calling errors and SNP calling errors [49]. Only unique alignments were considered and clusters were only selected if the read depth was between 4 and 40.

After read mapping and SNP calling (Figure 1), nucleotide diversity 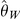 was estimated for each window according to a “modified Watterson estimator” based on the number of segregating sites *S*. Several estimators of this kind have been proposed for individual sequencing [28], [50], [51] which do not take into account the effect of sequencing pooled DNA. We have developed a “modified Watterson estimator” which is a Maximum Composite Likelihood estimator for pooled sequencing and is presented in equation 1, where *n_0_* is the sample size as measured by the number of independent chromosomes, *n_s_* (*i*) and *L(i)* are the read depth and length of the *ith* cluster, and *S* is the total number of segregating sites; *pSNP* is the consensus quality generated by MAQ [49], *a_j_* is the sum of *1/i* from *1* to *j-1*, *p_c_* is the probability that a set of chromosomes randomly extracted (with repetitions) from *n_o_* possible origins contains precisely *j* different chromosomes (equation 2), and *k* is the derived allele frequency.
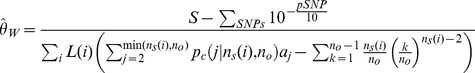
(1)


The second term in the denominator of equation 1, is applying a correction for sequencing and SNP calling errors, using 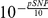 which is the probability that a SNP is not a true SNP according to the SNP quality estimator of MAQ [49] (for example, a value of *pSNP* = 20 means that the error probability is 1%, *pSNP* = 30 means 0.1%, and so on). In the denominator, 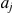 is weighted for the probability that in *j* sequences, we have *j* chromosomes (equation 2) and is corrected for the bias against rare variants in the SNP frequency spectrum of the data similar to the approach of Achaz [52].
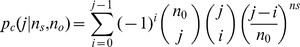
(2)


A dynamic algorithm was programmed in *C* by the authors to estimate 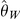. R scripts were developed to perform the required statistical analysis (http://cran.r-project.org/).

An error model was applied to infer the rate of false SNPs from the alignment output. The model predicts the relation 
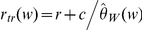
 where 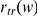 and 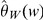 are the observed transition rate and observed variability for the *w* ^th^ window, *r* is the expected transition rate according to Amaral and colleagues [34] and *c* is the product between the base error rate and the difference between the transition rate for sequencing errors and the expected transition ratio. This model was fitted to the consensus sequence, in order to establish the best thresholds for further filtering for alignment quality (results not shown). Final calculations of 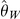 were performed after minimizing the rate of false SNPs by applying the following rules to the alignment output. Bases from positions 1 to 16 were selected for a *pSNP* larger or equal than 5, bases from positions 17 to 20 were selected for a *pSNP* larger or equal than 22, bases from positions 21 to 25 were selected for a *pSNP* larger or equal than 30, bases from positions 26 to 29 were selected for a *pSNP* larger or equal than 35 and bases from positions 31 to 33 were selected for a *pSNP* larger than or equal to 40.

### Confidence intervals for 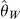


In order to identify candidate genomic regions that are under selection, the statistical uncertainty of our estimates (confidence intervals) was quantified by performing neutral coalescent simulations with recombination. Since for all chromosomes the average 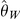 ranged from 0.001 to 0.0022 across breeds, we performed simulations with 1000 iterations each using MaCS [53] with parameters 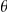  = 0.001 and 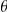  = 0.0022 and assuming levels of population recombination rate of 0.0015 and 0.002 respectively, similar to those used in previous studies [54]. For each simulation, a particular window of 500 Kb was simulated, sampled and 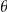 was estimated using equation 1. The variance for a generic value of 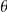 (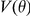) was then obtained by linear interpolation of the variance between the simulated values 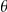  = 0.001 and 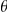  = 0.0022. A local average of 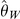 was estimated from the data over a sliding process covering 60 windows and excluding the 20 windows with the highest and lowest values in order to take into account regional differences in the levels of variability associated with regions of high and low recombination. Finally, confidence intervals with 95% confidence level were centered around the local average of 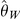 with width 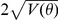. The procedure above was repeated for all windows in the data. Perl and R (http://cran.r-project.org/) scripts were developed by the authors to sample simulated windows according to the observed data and to estimate the confidence intervals.

### Modified estimator of genetic differentiation

The global *F_ST_* statistic for multiples sites defined by Nei [26] was modified in order to take into account pooled GA sequencing, sequence and consensus errors and ascertainment bias against singletons. For each pair of sampled populations, processed reads were selected in order that, only reads corresponding to the same genomic regions were used to access 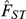 using the following equation,
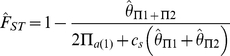
(3)where 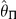 is the average nucleotide diversity between sequences coming from different sampled populations and *c_s_* is a factor that corrects for ascertainment bias towards singletons (Text S1). The pairwise nucleotide diversity between each population pair (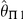 and 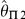) was estimated according to the following equation,
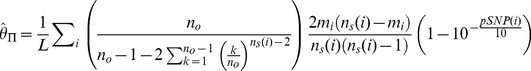
(4)where *i* runs over all SNPs in the considered window. The term in the first bracket corrects for pooling and singleton removal while the term in the last bracket corrects for the probability of false SNPs. *F_ST_* was estimated per genomic windows of 500 Kb and the significance of *F_ST_* values was estimated by a permutation test (1000 permutations per population pair). Genomic regions with *F_ST_* values in the 95% quartile with significant *p*-values (<0.05) were considered for further analysis as an ad hoc criteria used to select regions under selection.

### Tests of Means

Differences between the level of nucleotide diversity between chromosomes and between chromosome ends and telomeres were tested using the Wilcox test function in R (http://cran.r-project.org/). Centromeric coordinates were obtained in the pig map (version 1009 - http://pre.ensembl.org/Sus_scrofa_map/Location/Genome?r=2:6758451-7077409). Chromosome ends corresponded to windows in the two extremes of the chromosomes (5% of total number of windows).

### Functional enrichment analysis

We used the annotation for *Sus scrofa* assembly 8 available from pre-Ensembl (ftp://ftp.sanger.ac.uk/pub/S_scrofa/assemblies/PreEnsembl_Sscrofa8), generated by orthologous comparisons with human transcripts. Genes were considered to be overlapping when a genomic region of interest and the gene position were contained inside or partially inside the boundaries of the genomic region. The human Ensembl gene IDs were used to extract human Entrez gene IDs and protein family IDs by querying the Ensembl database (http://www.ensembl.org/) via the R package, biomaRt [55]. Using AnnotationDbi, a customized annotation R package was built using the Entrez gene IDs [56]. The GOstats package [57] was used to analyze enrichment in GO terms and KEGG pathways (http://www.geneontology.org/; http://www.genome.jp/kegg/pathway.html). Within the GOstats package a conditional hypergeometric test algorithm (Benjamini-Hochberg procedure) was applied for correction of multiple testing. The conditional hypergeometric test determined whether a GO term/KEGG pathway was significant when there was evidence beyond that provided by its significant children. Only the enriched GO term/KEGG pathway with raw *p*-values<0.05, were used for further interpretation in this study.

## Supporting Information

Figure S1 Summary statistics for all the SNPs identified in Large White.
(0.03 MB PDF)

Figure S2 Fst values (A) and p-values frequency (B) presented by breed pair.
(0.26 MB PDF)

Table S1 Descriptions of breeds sampled.
(0.14 MB PDF)

Table S2 Summary of genes related to neuron function and that overlap with genomic regions with significant low θ_W_. Summary of genes related to growth, muscle development, metabolism and disease that overlap with genomic regions with significant low θ_W_.
(0.08 MB PDF)

Table S3 Summary of genes related to growth, muscle development, metabolism and disease that overlap with genomic regions with significant low θ_W_.
(0.16 MB PDF)

Text S1 Detailed description of measures of polymorphism and genetic differentiation.
(0.11 MB PDF)
