# Supplementary material for: Genome-Wide Footprints of Pig Domestication and Selection Revealed through Massive Parallel Sequencing of Pooled DNA
Source: PLoS One. 2011 Apr 4;6(4):e14782. doi: 10.1371/journal.pone.0014782 (PMC3070695; doi:10.1371/journal.pone.0014782)

Figure S1 – Summary statistics for all the SNPs identified in Large White. (A) SNP counts per position in the GA sequences. Red bars represent transitions and blue bars represent transversions. (B) Histogram showing counts of minor allele.

(A)

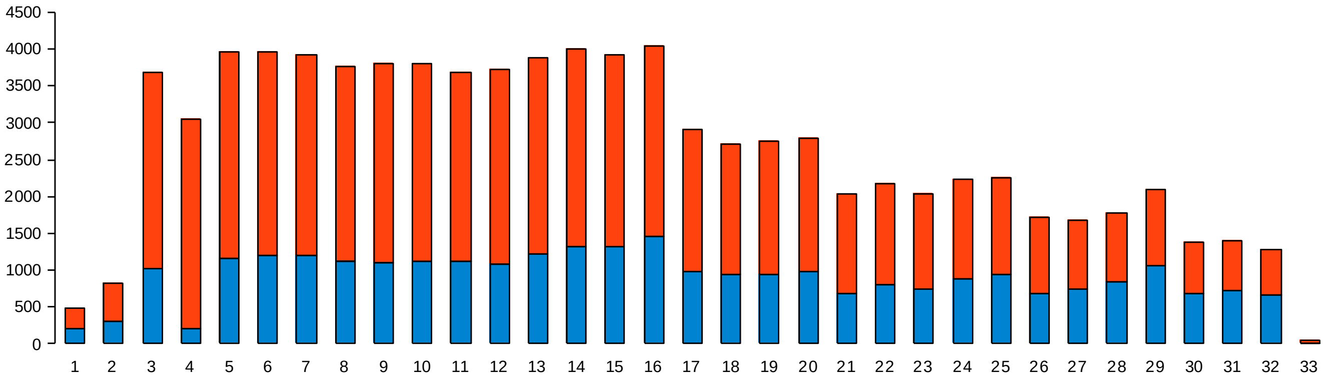

(B)

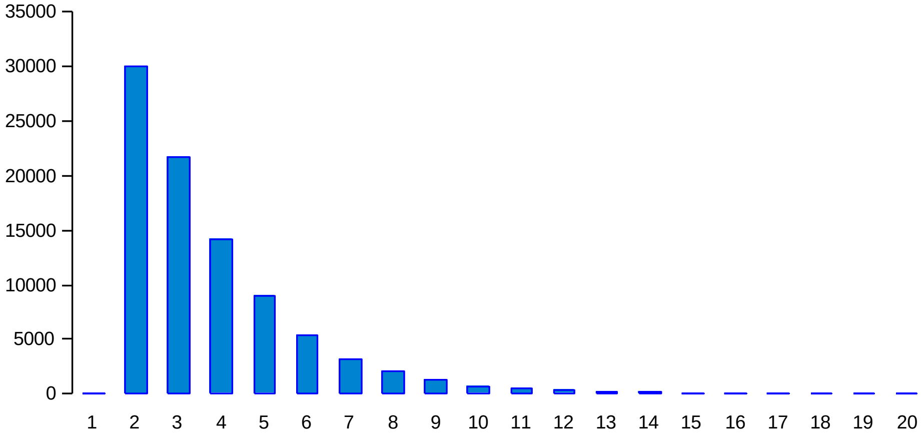

Supplement: Figure S1 — Summary statistics for all the SNPs identified in Large White. (0.03 MB PDF) [file pone.0014782.s001.pdf]
