# Supplementary material for: Genome-Wide Footprints of Pig Domestication and Selection Revealed through Massive Parallel Sequencing of Pooled DNA
Source: PLoS One. 2011 Apr 4;6(4):e14782. doi: 10.1371/journal.pone.0014782 (PMC3070695; doi:10.1371/journal.pone.0014782)
Supplement: Figure S2 — Fst values (A) and p-values frequency (B) presented by breed pair. (0.26 MB PDF) [file pone.0014782.s002.pdf]

**Figure S2** – $F_{st}$ values (A) and p-values frequency (B) presented by breed pair. (A) x axis is the chromosome from SSC1 to SSC18 and finally SSCX.

(A)

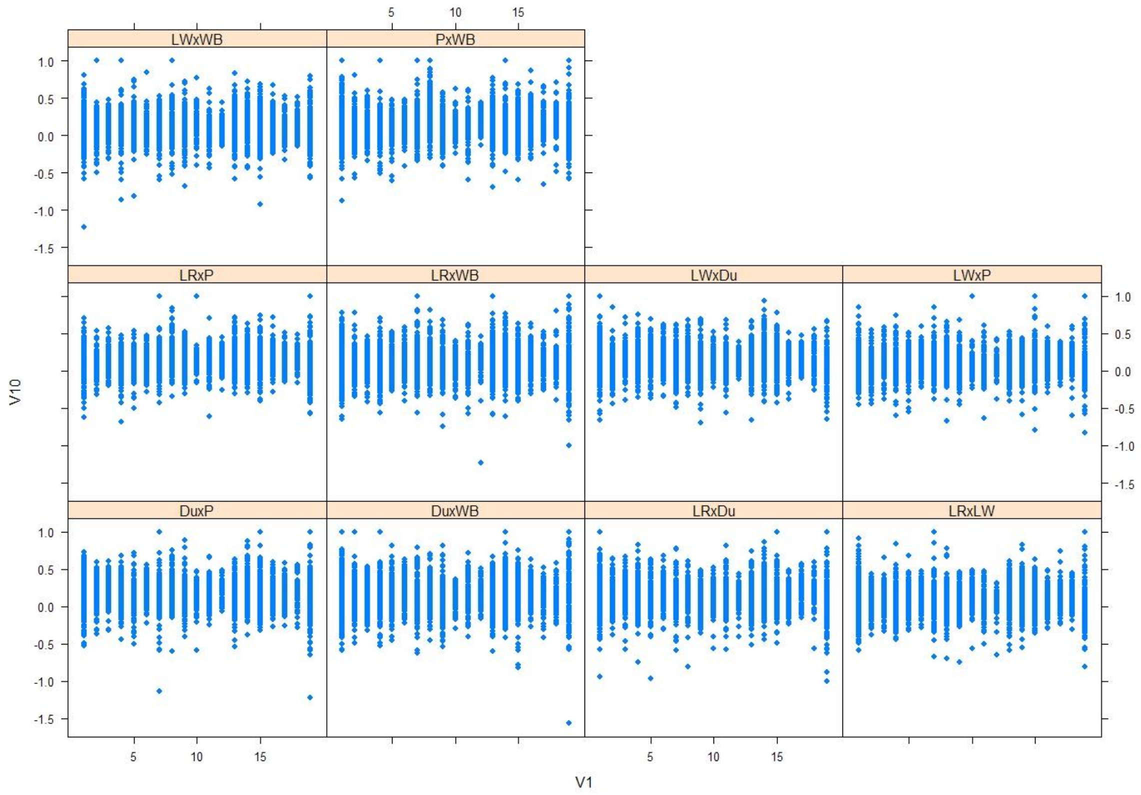

(B)

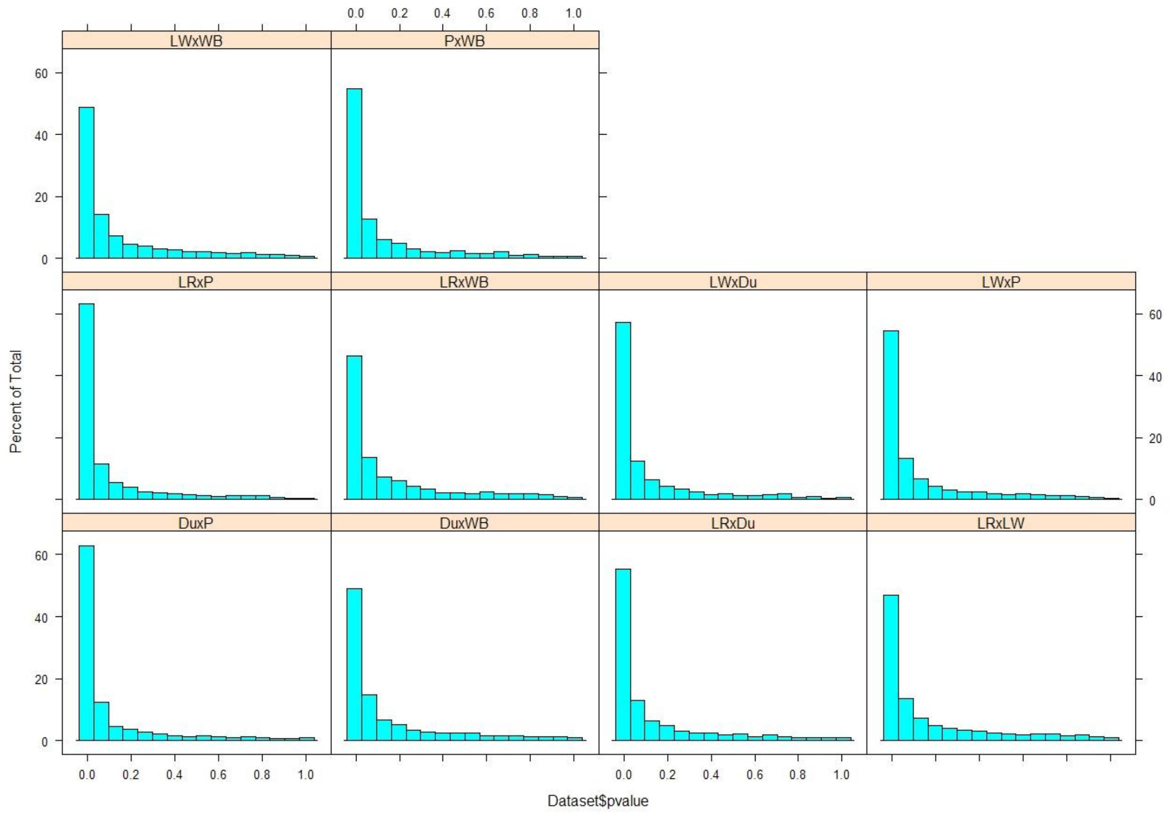
LWxWB
PxWB
LRxP
LRxWB
LWxDu
LWxP
DuxP
DuxWB
LRxDu
LRxLW
Percent of Total
Dataset$pvalue
0.0
0.2
0.4
0.6
0.8
1.0
0
20
40
60
