# Supplementary material for: Genome-Wide Footprints of Pig Domestication and Selection Revealed through Massive Parallel Sequencing of Pooled DNA
Source: PLoS One. 2011 Apr 4;6(4):e14782. doi: 10.1371/journal.pone.0014782 (PMC3070695; doi:10.1371/journal.pone.0014782)
Supplement: Table S1 — Descriptions of breeds sampled. (0.14 MB PDF) [file pone.0014782.s003.pdf]

Table S1- Description of breeds sampled

Breed	Breed history	Characteristics/Aptitudes	Major faults	Country of origin	Sample size
**Large White**	Origin in England late 1700s from a cross between white pigs from Yorkshire and Chinese pig from Canton	• Large litter sizes<br>• Large milk production<br>• Good maternal instincts<br>• Good feet and legs	• Poor ham development	Denmark, USA, The Netherlands	36
**Landrace**	Origin in Denmark from a cross between local wild boars and imported Large White pigs	• Large size<br>• Large eye muscle<br>• Good carcass quality<br>• Rapid growth<br>• Higher weight on weaning	• Leg weakness<br>• Splay legs<br>• Nervous disorders (porcine stress syndrome)	Denmark, USA, The Netherlands	29
**Pietrain**	Originated from Pietrain in Belgium, exact origin is unknown	• Legs with extreme muscular development<br>• High proportion of lean to fat<br>• Desired for fresh meat production<br>• Used as sire lines	• High mortality due to high frequency of a mutation in the Hal gene<br>• Poorer meat quality	The Netherlands, USA	23
**Duroc**	Unknown origin. By ~1800 several red breeds were developed in USA, including the Duroc of New York	• Large size<br>• Heavy carcass production<br>• Use as terminal sire line when crossed with Large White×Landrace sows for bacon production	• Bad maternal instincts<br>• Lower litter size<br>• Late maturing type	Denmark, USA, The Netherlands	34
