# Supplementary material for: Genome-Wide Footprints of Pig Domestication and Selection Revealed through Massive Parallel Sequencing of Pooled DNA
Source: PLoS One. 2011 Apr 4;6(4):e14782. doi: 10.1371/journal.pone.0014782 (PMC3070695; doi:10.1371/journal.pone.0014782)
Supplement: Table S2 — Summary of genes related to neuron function and that overlap with genomic regions with significant low θW. Summary of genes related to growth, muscle development, metabolism and disease that overlap with genomic regions with significant low θW. (0.08 MB PDF) [file pone.0014782.s004.pdf]

Table S2- Summary of genes related to neuron function and that overlap with genomic regions with significant low $\hat{\theta}_W$.

**Position (Mb)**	**Breed**	$\hat{\theta}_W$ **(x10^{-4})**	**95% CI (x10^{-3})**	**Overlapping gene**
SSC12:15-15.5	Landrace	6.45	1.1-2.7	*PPP1R1B*
	Duroc	5.69	1.0-2.7	
	Large White	8.0	1.1-2.9	
SSC2:94.5-95	Landrace	9.81	1.4-3.8	*LRRTM2*
	Pietrain	10.4	1.4-3.5	
	Large White	13.6	1.4-4.0	
SSC12:8.5-9	Landrace	6.8	1.0-2.7	*CACGN4*
	Pietrain	10.5	1.2-2.6	
SSC10:30.5-31	Landrace	5.2	0.9-2.8	*LINGO2*
SSC11:39-39.5	Pietrain	4.6	0.8-2.5	*CLN5*
	Large White	5.9	0.7-2.6	
SSC14:54-54.5	Pietrain	3.5	0.7-2.0	*HRK*
	Duroc	4.5	0.4-1.9	
SSC5:19-19.5	Large White	6.2	0.9-3.2	*KIF5A*
SSCX:6-6.5	Pietrain	1.9	0.5-2.2	*GPM6B*
	Large White	1.8	0.3-2.4	
SSC6:19-19.5	Landrace	5.8	0.8-2.3	*CACNG6*
	Duroc	8.1	0.9-2.7	
	Landrace	5.8	0.8-2.3	*CACGN8*
	Duroc	8.1	0.9-2.7	
	Landrace	10.5	1.2-2.9	*CACGN7*

	Duroc	8.1	0.9-2.7	
SSC3:35.5-36	Landrace	3.2	0.8-2.3	*LRRTM4*
	Pietrain	4.9	0.5-1.9	
SSC12:35.5-36	Landrace	10.5	1.2-2.9	*ENO2*
SSC7:58-58.5	Duroc	4.1	0.5-2.3	*AP3B2*
SSCX:61-61.5	Landrace	0	0.1-1.8	*DCX*
SSC4:44-44.5	Landrace	3.7	0.5-2.1	*NECAB1*
	Large White	3.6	0.4-2	
SSCX:6-6.5	Pietrain	2.0	0.4-2.2	*GPM6B*
	Duroc	2.1	0.2-1.9	
SSC7:81-81.5	Landrace	4.2	0.5-2.2	*CPNE6*
	Large White	7.0	0.7-2.8	
SSC14:127-127.5	Large White	5.7	0.7-2.5	*SMNDC1*
	Duroc	6.6	0.7-2.4	
SSC1:228-228.5	Duroc	2.8	0.4-2.1	*NR4A3*
SSC5:2.5-3	Large White	9.7	1.0-3.5	*SEPT3*
SSC3:2.5-3	Pietrain	11.9	1.2-2.9	*LRCH4*
SSC7:104.5-105	Duroc	7.4	0.7-2.8	*PNMA1*
SSC7:79-79.5	Pietrain	6.6	0.7-2.4	*NOVA1*
SSC1:	Landrace			*TMOD2*

Table S2- Summary of genes related to brain function and that overlap with genomic regions with significant low $\hat{\theta}_W$.

**Position (Mb)**	**Breed**	$\hat{\theta}_W$ **(x10^{-4})**	**95% CI (x10^{-3})**	**Overlapping gene**
SSC3:43.5-44	Landrace	2.2	0.8-2.7	*TTBK1*
	Pietrain	7.1	0.9-2.7	
SSC2:44.5-45	Landrace	2.0	0.6-2.2	*TMEM59L*
	Large White	4.9	0.6-2.3	
	Pietrain	6.0	0.6-1.8	
SSC12:13-13.5	Landrace	7.1	1.0-2.6	*KCNH4*
	Duroc	8.1	1.0-2.6	
SSC4:81.5-82	Duroc	1.4	0.4-2.1	*BRP44*
	Large White	3.6	0.4-2.2	
	Landrace	4.0	0.4-1.9	
SSC2:22.5-23	Duroc	4.3	0.7-2.6	*BDNF*
SSC13:132-132.5	Duroc	6.0	0.8-2.9	*DYRK1A*
SSC12:24-24.5	Duroc	7.6	0.9-2.6	*SEPT4*
	Large White	10	1.1-2.8	
SSC13:23.5-24	Pietrain	6.5	0.8-2.3	*CSPG5*
	Duroc	5.8	0.7-2.6	
SSC7:43.5-44	Pietrain	7.1	0.9-2.6	*TTBK1*
SSC8:54-54.5	Duroc	5.6	0.7-2.9	*POU4F2*
SSC15:53.5-54	Pietrain	0	0.2-1.1	*TBR1*
SSC15:56.5-57	Large White	0	0.2-1.5	*SCN1A, SCN2A,*
	Duroc	2.2	0.4-1.9	*SCN3A*

SSC2:44.5-45	Large White	4.9	0.6-2.4	*TMEM59L*
	Pietrain	6.0	0.6-1.9	
SSC5:3-3.5	Pietrain	9.7	1.1-2.9	*L3MBTL2*
SSC5:10.5-11	Duroc	6.0	0.7-2.7	*RIC8B*
SSC5:5.5-6	Large White	8.7	0.9-3.3	*BAIAP2L2*
SSC9:104.5-105	Duroc	8.8	0.9-3.0	*VWC2*
SSCX:23.5-24	Pietrain	1.5	0.2-1.4	*TMEM47*
SSC7:81.5-82	Landrace	6.0	0.5-2.3	*SLC22A17*
