# Supplementary material for: Genome-Wide Footprints of Pig Domestication and Selection Revealed through Massive Parallel Sequencing of Pooled DNA
Source: PLoS One. 2011 Apr 4;6(4):e14782. doi: 10.1371/journal.pone.0014782 (PMC3070695; doi:10.1371/journal.pone.0014782)
Supplement: Table S3 — Summary of genes related to growth, muscle development, metabolism and disease that overlap with genomic regions with significant low θW. (0.16 MB PDF) [file pone.0014782.s005.pdf]

Table S3- Summary of genes related to growth, muscle development, metabolism and disease that overlap with genomic regions with significant low $\hat{\theta}_W$.

Position(Mb)	Breed	$\hat{\theta}_W$ (x10^{-4})	95%CI(x10^{-3})	Overlapping gene
VEGF signaling pathway				
SSC7:35.5-36	Duroc	9.64	0.96-3.4	MAPK14
	Pietrain	8.05	1-2.9	
SSC12:37-37.5	Duroc	9.04	1.1-2.8	PIK3R5
	Wild boar	6.43	0.8-2.5	
SSC4:104-104.5	Landrace	7.00	1-3.1	KRAS
	Large white	6.94	0.7-2.75	
	Pietrain	7.69	0.9-2.6	
SSC7:81-81.5	Landrace	4.23	0.5-2.2	NFATC4
	Large white	7.05	0.7-2.7	
SSC17:39-39.5	Large white	7.76	1-3.2	PLCG1
SSC6:19-19.5	Duroc	8.14	0.8-2.7	PRKCG
	Landrace	5.83	0.8-2.3	
SSC14:51.5-52	Landrace	4.25	0.7-2.4	MAPK1
	Large white	2.86	0.4-1.9	
	Pietrain	6.30	0.6-1.9	
SSC8:74.5-75	Pietrain	4.51	0.5-1.8	PLA2G12A
SSC12:2-2.5	Large white	11.41	1.2-3.1	SPHK1
	Pietrain	11.68	1.2-2.6	
SSC9:108-108.5	Duroc	6.92	1-3.2	MAPKAPK2
	Large white	8.41	0.8-2.8	
	Pietrain	6.71	1.1-2.8	
Arachidonic acid metabolism pathway				
SSC8:75.5-76	Duroc	9.94	1-3.5	CYP2U1
	Landrace	10.3	1-3.5	
SSC14:95.5-96	Landrace	7.35	0.8-2.4	ALOX5
	Large white	3.69	0.5-2.1	
SSC17:34.5-35	Landrace	3.28	0.8-2.5	GGT7
	Large white	9.65	0.9-3.1	
	Pietrain	4.56	0.9-2.5	
SSC17:45-45.5	Pietrain	8.18	0.8-2.3	PTGIS
SSC13:130.5-131	Landrace	8.86	0.9-2.9	CBR3
	Pietrain	10.40	1.1-2.8	
SSC1:256-256.5	Landrace	3.18	0.8-3.1	PTGES
	Large white	6.75	0.8-3.23	
	Pietrain	9.95	1.1-3.1	
Mapk signaling pathway				
SSC10:9.5-10	Duroc	8.15	1.1-3.5	DUSP10

	Landrace	8.51	1.3-3.5	
	Large white	10.48	1.1-3.4	
SSC14:52-52.5	Landrace	3.84	0.7-2.4	CRKL
	Wild boar	2.48	0.2-2.0	
SSC7:35.5-36	Duroc	9.64	0.9-3.4	MAPK14
	Pietrain	8.05	1.1-3	
SSC12:12.5-13	Large white	11.3	1.1-2.8	DUSP3
SSC14:127-127.5	Duroc	6.57	0.6-2.4	DUSP5
	Large white	5.7	0.7-2.5	
SSC14:118-118.5	Landrace	7.33	0.7-2.4	FGF8
	Pietrain	6.6	0.7-2.1	
	Wild boar	5.5	0.5-2.7	
SSC16:20-20.5	Large white	8.94	0.9-3.2	FGF10
SSC14:137.5-138	Pietrain	8.8	0.9-2.6	FGFR2
SSC1:145-150	Duroc	8.1	0.9-3.3	MAP3K7IP2
	Landrace	9.8	1-3.3	
SSC3:18-18.5	Duroc	6.1	0.6-2.2	MAPK8IP3
	Landrace	4.2	0.9-2.6	
	Large white	3.7	0.8-2.5	
	Pietrain	1.1	1-2.5	
	Wild boar	5.4	0.5-2.5	
SSC7:106-106.5	Duroc	4.8	0.7-2.8	FOS
	Large white	5.6	0.6-2.6	
SSC6:17.5-18	Landrace	6.3	0.8-2.3	FGF21
SSC12:2.5-3	Pietrain	9.6	1.2-2.6	GRB2
SCC4:104-104.5	Landrace	7.0	1-3.1	KRAS
	Large white	6.94	0.7-2.7	
	Pietrain	7.69	0.9-2.6	
SSC7:81-81.5	Landrace	4.2	0.6-2.2	NFATC4
	Large white	7.0	0.7-2.8	
SSC13:28.5-29	Duroc	6.4	0.7-2.5	CACNA2D3
	Landrace	5.2	0.7-2.3	
	Large white	5.7	0.6-2.3	
SSC6:19-19.5	Duroc	8.1	0.8-2.7	PRKCG
	Landrace	5.8	0.8-2.3	
SSC14:51.5-52	Landrace	4.2	0.7-2.4	MAPK1
	Large white	2.9	0.4-1.9	
	Pietrain	6.3	0.6-1.9	
6:19-19.5	Duroc	8.1	0.8-2.7	CACNG8
	Landrace	5.8	0.8-2.7	
6:19-19.5	Duroc	8.1	0.8-2.7	CACNG7
	Landrace	5.8	0.8-2.3	

6:19-19.5	Duroc	8.14	0.8-2.7	CACNG6
	Landrace	5.83	0.8-2.7	
7:43.5-44	Landrace	2.23	0.7-2.7	SRF
	Pietrain	7.2	0.9-2.7	
13:28-28.5	Landrace	5.38	0.7-2.3	CACNA1D
	Large white	5.62	0.6-2.3	
12:8.5-9	Landrace	6.8	1.1-2.7	CACNG1
	Pietrain	1.1	1.2-2.6	
8:74.5-75	Pietrain	4.5	0.5-1.8	PLA2G12A
9:108-108.5	Duroc	6.9	1-3.2	MAPKAPK2
	Large white	8.4	0.8-2.9	
	Pietrain	6.7	1.1-2.8	
p53 signaling pathway				
SSC9:40-40.5	Duroc	4.34	0.5-2.1	CHEK1
	Landrace	3.70	0.8-2.5	
	Large white	4.49	0.5-2.2	
	Pietrain	4.06	0.7-2.1	
SSC4:33-33.5	Pietrain	4.81	0.5-1.8	RRM2B
SSC15:48.5-49	Duroc	3.47	0.4-2.1	RPRM
	Large white	0.0	0.1-1.4	
SSC14:105.5-106	Duroc	3.32	0.5-2.2	PTEN
SSC4:39.5-40	Duroc	5.13	0.6-2.5	CCNE2
SSC9:40-40.5	Duroc	4.34	0.5-2.1	EI24
	Landrace	3.70	0.8-2.5	
	Large white	4.49	0.5-2.2	
	Pietrain	4.06	0.7-2.1	
Gnrh signaling pathway				
SSC7:35.5-36	Duroc	9.64	0.9-3.3	MAPK14
	Pietrain	8.05	1-2.9	
SSC7:81-81.5	Landrace	4.23	0.5-2.2	ADCY4
	Large white	7.05	0.7-2.8	
SSC2:20-20.5	Pietrain	6.03	0.9-2.5	FSHB
SSC17:53.5-54	Duroc	8.17	0.8-2.7	GNAS
	Wild boar	3.94	0.5-2.6	
SSC4:104-104.5	Landrace	7.00	1-3.2	KRAS
	Large white	6.94	0.7-2.8	
	Pietrain	7.69	0.9-2.6	
SSC14:51.5-52	Landrace	4.25	0.7-2.4	MAPK1
	Large white	2.86	0.4-1.9	
	Pietrain	6.30	0.6-1.9	
SSC13:28-28.5	Landrace	5.38	0.6-2.3	CACNA1D
	Large white	5.62	0.6-2.3	

SSC8:74.5-75	Pietrain	4.51	0.5-1.8	PLA2G12A
SSC14:79-79.5	Landrace	1.54	0.6-2.2	CAMK2G
Bladder cancer pathway				
SSC13:25-25.5	Duroc	4.85	0.6-2.6	RASSF1
	Landrace	7.44	0.7-2.6	
SSC17:33.5-34	Landrace	5.84	0.8-2.5	E2F1
SSC12:15-15.5	Duroc	5.69	1-2.7	ERBB2
	Landrace	6.45	1-2.6	
	Large white	8.01	1-2.9	
SSC19:7-7.5	Pietrain	2.65	0.4-2.2	FIGF
SSC4:104-104.5	Landrace	7.00	1-3.2	KRAS
	Large white	6.94	0.7-2.8	
	Pietrain	7.69	0.9-2.6	
SSC14:51.5-52	Landrace	4.25	0.7-2.4	MAPK1
	Large white	2.86	0.4-1.9	
	Pietrain	6.30	0.6-1.9	
SSC15:30-30.5	Landrace	4.53	0.5-2.1	VEGFC
	Wild boar	0.0	0.4-2.7	
