# Supplementary material for: Genome-Wide Footprints of Pig Domestication and Selection Revealed through Massive Parallel Sequencing of Pooled DNA
Source: PLoS One. 2011 Apr 4;6(4):e14782. doi: 10.1371/journal.pone.0014782 (PMC3070695; doi:10.1371/journal.pone.0014782)
Supplement: Text S1 — Detailed description of measures of polymorphism and genetic differentiation. (0.11 MB PDF) [file pone.0014782.s006.pdf]

Supplementary Text S1

The modified Watterson estimator that accounts for DNA pooling, sequencing errors and ascertainment bias for singletons is

$$\hat{\theta}_W = \frac{S - \sum_s 10^{-\frac{pSNP(s)}{10}}}{\sum_i L(i) \left(\sum_{j=2}^{\min(n_s(i),n_0)} P_c(j|n_s(i), n_0) a_j - \sum_{k=1}^{n_0-1} \frac{n_s(i)}{n_0} \left(\frac{k}{n_0}\right)^{n_s(i)-2} \right)}$$

where S is the number of segregating sites that are not singletons, $pSNP(s)$ is the Phred-scaled probability that the sth SNP is a sequencing error, $n_s(i)$ and $L(i)$ are the read depth of the ith cluster of sequences (that is, a contiguous region with constant read depth) and n_0 is the number of independent chromosomes in the sample (which is twice the sample size). $P_c(j|n_s, n_0)$ is the probability that the output of n_s random extractions (with replacement) from a box of n_0 different objects contains exactly j different objects. An explicit formula for $P_c(j|n_s, n_0)$ is

$$P_c(j|n_s, n_0) = \sum_{i=0}^{j-1} (-1)^i \binom{n_0}{j} \binom{j}{i} \left(\frac{j-i}{n_0}\right)^{n_s}$$

The estimator for pairwise nucleotide diversity which includes corrections for sequencing errors and absence of singletons is

$$\hat{\theta}_\Pi = \frac{1}{L} \sum_i \left(\frac{n_0}{n_0 - 1 - 2\sum_{k=1}^{n_0-1} (k/n_0)^{n_s(i)-2}} \right) \frac{2m_i(n_s(i) - m_i)}{n_s(i)(n_s(i)-1)} \left(1 - 10^{-\frac{pSNP(i)}{10}}\right)$$

where m_i is the minor allele count of the ith SNP.

The formula for F_{ST} between two populations using the definition of Nei (Molecular Evolutionary Genetics, 1987) is

$$\hat{F}_{ST} = 1 - \frac{\hat{\theta}_{\Pi 1} + \hat{\theta}_{\Pi 2}}{2\Pi_a + c_s(\hat{\theta}_{\Pi 1} + \hat{\theta}_{\Pi 2})}$$

where $\hat{\theta}_{\Pi 1}$ and $\hat{\theta}_{\Pi 2}$ are the nucleotide diversity estimators for the two populations, Π_a is the pairwise nucleotide diversity between sequences coming

from different populations and c_s is a correction factor given by

$$
\begin{aligned}
c_s = &\sum_{k=1}^{n_0^{(1)}+n_0^{(2)}-1} \frac{1}{k} \sum_{l=0}^{k} \frac{\binom{n_0^{(1)}}{k-l}\binom{n_0^{(2)}}{l}}{\binom{n_0^{(1)}+n_0^{(2)}}{k}} \times \\
&\times \Big\{ (y_2 - x_2)x_1y_1[y_1^{n_s^{(1)}-2} - x_1^{n_s^{(1)}-2}] + (y_1 - x_1)x_2y_2[y_2^{n_s^{(2)}-2} - x_2^{n_s^{(2)}-2}] + \\
&-(n_s^{(1)} + n_s^{(2)})x_1y_1x_2y_2[x_1^{n_s^{(1)}-2} + y_1^{n_s^{(1)}-2}][x_2^{n_s^{(2)}-2} + y_2^{n_s^{(2)}-2}] + \\
&+ 2x_1y_1x_2y_2[x_1^{n_s^{(1)}-2} - y_1^{n_s^{(1)}-2}][x_2^{n_s^{(2)}-2} - y_2^{n_s^{(2)}-2}] \Big\}
\end{aligned}
$$

with $x_1 = (k-l)/n_0^{(1)}$, $x_2 = l/n_0^{(2)}$, $y_1 = 1 - x_1$ and $y_2 = 1 - x_2$.

The above estimators $\hat{\theta}_W$, $\hat{\theta}_\Pi$ and $\Pi_a + c_s(\hat{\theta}_{\Pi 1} + \hat{\theta}_{\Pi 2})/2$ are unbiased estimators of θ (Ferretti, Ramos-Onsins and Perez-Enciso, personal communication).
